# Experience of undergraduate nursing students participating in artificial intelligence + project task driven learning at different stages: a qualitative study

**DOI:** 10.1186/s12912-024-01982-1

**Published:** 2024-05-08

**Authors:** Weijuan Kong, Yanhua Ning, Ting Ma, Fei Song, Yuxin Mao, Cailing Yang, Xinjin Li, Yahong Guo, Haiyan Liu, Jing Shi, Lingna Liu

**Affiliations:** School of Nursing, Ningxia Medical University, Yinchuan, Ningxia 750004 China

**Keywords:** Undergraduate nursing students, Artificial intelligence, Project task driven learning, Experience

## Abstract

**Background:**

Artificial intelligence is a growing phenomenon that will soon facilitate wide-scale changes in many professions, and is expected to play an important role in the field of medical education. This study explored the realistic feelings and experiences of nursing undergraduates participating in different stages of artificial intelligence + project task driven learning, and provide a basis for artificial intelligence participation in nursing teaching.

**Methods:**

We conducted face-to-face semi-structured interviews with nursing undergraduates participating in Nursing Research Course which adopts artificial intelligence + project task driven learning from a medical university in Ningxia from September to November 2023, to understand their experience of using artificial intelligence for learning and the emotional changes at different stages. The interview guide included items about their personal experience and feelings of completing project tasks through dialogue with artificial intelligence, and suggestions for course content. Thematic analysis was used to analyze interview data. This study followed the COREQ checklist.

**Results:**

According to the interview data, three themes were summarized. Undergraduate nursing students have different experiences in participating in artificial intelligence + project task driven learning at different stages, mainly manifested as diverse emotional experiences under initial knowledge deficiency, the individual growth supported by external forces during the adaptation period, and the expectations and suggestions after the birth of the results in the end period.

**Conclusions:**

Nursing undergraduates can actively adapt to the integration of artificial intelligence into nursing teaching, dynamically observe students’ learning experience, strengthen positive guidance, and provide support for personalized teaching models, better leveraging the advantages of artificial intelligence participation in teaching.

## Background

ChatGAi, an artificial intelligence (AI) generated content (AIGC) model developed by OpenAI, has attracted world-wide attention for its capability of dealing with challenging language understanding and generation tasks in the form of conversations [1]. At the same time, Baidu in China also launched a generative dialogue application based on the big model technology: ERNIE Bot [2]. Both of them were intelligent chat bots built on the foundation of AI technology, which can automatically generated answers based on user input content and intentions. They are powerful in information combination and interaction, so can produce “New” text content by processing and analyzing huge data, and then present it to the users, and have the potential to become “knowledge assistants” for educators truly [1, 3].

Large language models can serve as virtual teaching assistants, providing students with detailed and relevant information and perhaps eventually interactive simulations [4]. Integrating generative AI into more medical scenarios can further improve the efficiency and quality of medical services, giving doctors more time to communicate with patients and implement personalized health management [5]. It provides outstanding prospects for immersive and personalized learning experiences, strengthening the effectiveness and efficiency of nurse education for remote patient monitoring [6]. At present, researches on AI represented by ChatGAi mainly focused on macro description of the application prospects in a certain professional field [7, 8], and applied it to language translation, programming, and testing for comparison with manual completion of a certain task [9, 10]. There are some studies have also explored students’ usage intentions and the opinions of professional teachers [11]. Nursing education is critical for nurses to deliver quality health care; incorporating AI into education can enhance the learning process and better equip nurses for their health care roles [12]. For nursing related teaching content, clinical nurses are the main focus, and the attention was paid to the application analysis of nursing practice [13], lacking attention to nursing students on campus.

Under this background, the research introduced the generative AI application tools represented by ChatGAi and ERNIE Bot into the teaching of undergraduate Nursing Research Course, carried out practical content teaching in the way of “AI + project task driven learning”. The project task in this study was to provide students with six different project issues based on the teacher’s teaching reform project and teaching content. Each course revolved around different aspects of the project issues, and at the end of the course, each project issue can form a paper. According to the course schedule, we conducted semi-structured interviews with students participating in this course; understand their real feelins and experience of participating in learning, in order to better improving teaching.

Therefore, this study adopted qualitative study to explore the changes in students’ inner experiences when participating in AI + project task driven learning, from the perspective of nursing undergraduates and different time points, aimed to provide reference for the further application of AI in nursing teaching and other teaching, and adapt to the teaching reform under the background of intelligence.

## Methods

### Design

This study was based on qualitative study design to understand the experience of using artificial intelligence for learning with nursing undergraduates. The reporting of the qualitative results adhered to the COREQ guidelines. We adopted the thematic analysis to identify the themes at different stages [14].

### Course arrangement

**Fig. 1:**
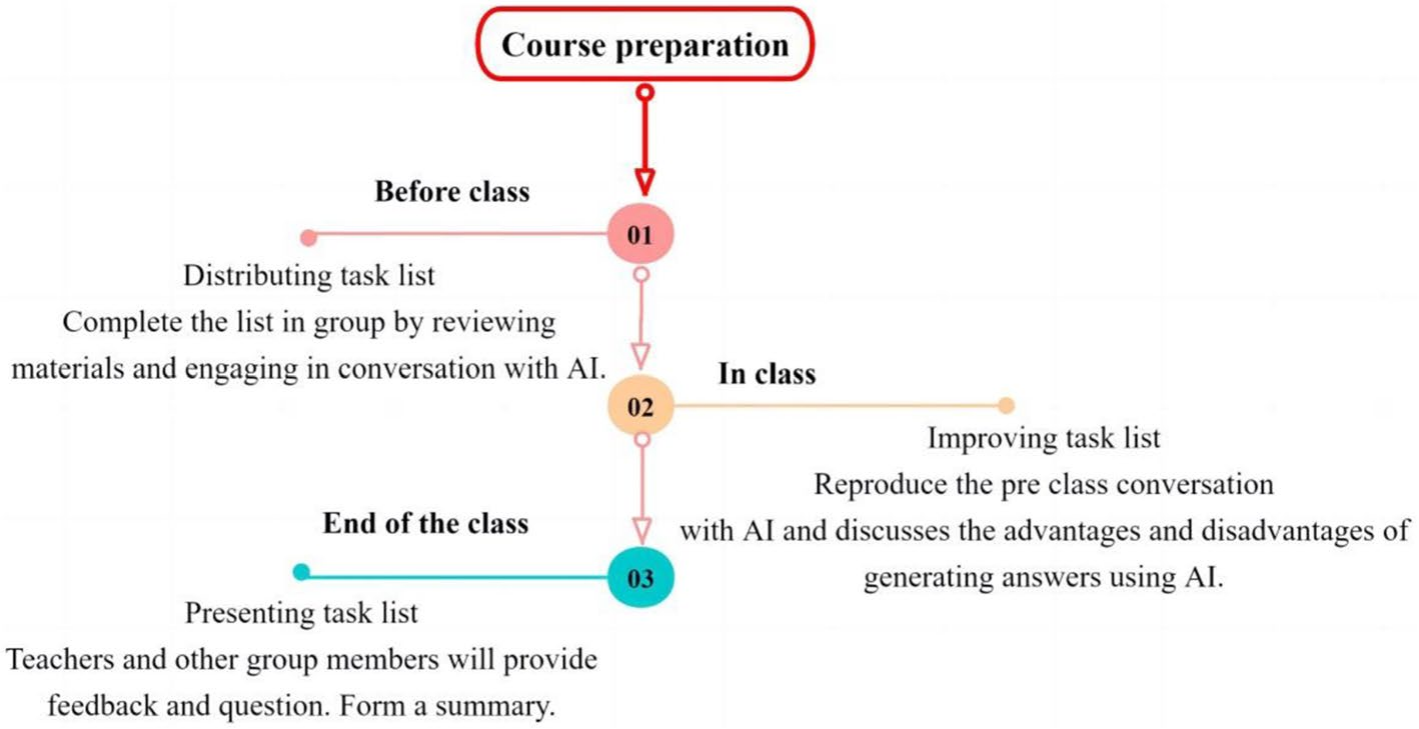
Process arrangement for each experimental course

1. Assistant training: Prior to the start of the course, six graduate students from grades two and three are selected as teaching assistants for this course, the instructors provide them with unified training on Nursing Research Course and the use of AI. The project task for each group is derived from the content of the educational reform project by the instructors. The teaching assistants complete the task list in advance and communicate with the instructors at any time if there are any problems.
2. Class practice: This course mainly focuses on the teaching content of undergraduate Nursing Research Course, including group topic selection and argumentation, research design determination, sample size calculation and sampling methods, research tools and data collection methods, data analysis and paper writing. The project tasks have been determined before class, and in the first class, the group leaders draw lots to determine the content of the issues, and complete the prescribed teaching content in order according to the course schedule.
3. Class content: the general process arrangement for each experimental course is as Fig. 1.

### Sample recruitment

The participants were the third year students from the school of nursing participated in the Nursing Research Course. The experimental course used an “AI + project task driven learning” approach to learn. A purposive sampling approach was used to recruit participants who met the principle of maximum differentiation as the interview subjects. According to the class performance and research purpose, we communicated with 16 students through face-to-face interview and wechat contact. Before the research, we explained the purpose of the study, the methodology, the process of data collection, and the fact that they can withdraw from the study at any time. All interviewees voluntarily participated and signed an informed consent form. The sample size of the study was subject to the fact that no new information appears in the interview data. Finally, 14 students participated in this study. Recruitment and data collection occurred between September and December 2023.

### Data collection

Semi-structured interviews were conducted with fourteen undergraduate nursing students participating in Nursing Research Course which adopts AI + project task driven learning to understand their experience of using artificial intelligence for learning. The interview guide included items about their personal experience and feelings of completing project tasks through dialogue with artificial intelligence, and suggestions for course content. Thematic analysis was used to analyze interview data.

The principal investigators in this study as the teaching assistants of the course, and established a good contact with nursing undergraduates. We used a semi-structured interview before, during, and after the course, using the interview script shown in Table 1.

**Table 1:** Interview script

Interview questions
What is your most intuitive experience of participating in AI + project task driven learning?
What pleasant or unpleasant experiences did you have during your participation in AI + project task driven learning?
How did you complete classroom tasks in the process of artificial AI + project task driven learning?
How about participation?
What do you think is the biggest difficulty you face when participating in AI + project task driven learning?
What benefits do you expect to gain from participating in AI + project task driven learning?
What are your opinions and suggestions on the current implementation of AI + project task driven learning?

Probing questions not included

The above questions were not unique, and adjustments would be made during the interview process based on the answers provided by the interviewees. Before each interview, we contacted with the interviewee first, informed them of the main research content, and determined the interview location and time in advance. Prepared an informed consent form, interview script, and general situation survey form for the interview. Before the interview, informed the students again of the purpose, methods, and confidentiality principles of this study. After obtaining the informed consent of the participants, started the interview. During the interview, the interviewee was asked relevant questions about AI + project task driven learning according to the interview script. After obtaining the consent of the participants, the entire process was recorded using a mobile phone. The emotional and behavioral responses of the interviewees were be carefully observed and recorded. Each interview was jointly completed by two researchers, responsible for the interview and recording.

### Data analysis

Interviews were conducted in Chinese, and then translated into English by two researchers, and finally verified by a doctor. The main phases of thematic [14]: familiarizing yourself with your data, generating initial codes, searching for themes, reviewing themes, defining and naming themes, producing the report. We supported our findings with quotes from individual interviews.

### Quality control

1. Before class, the Nursing Research teaching group and teaching assistants work together to prepare the lesson, including clarifying the course content, teaching methods, and responsibilities of the teachers and teaching assistants.
2. The task list is issued in advance for each experimental class. Before the class, the students have an autonomous dialogue with AI such as ChatGAi and ERNIE Bot, and carry out group discussion to complete the task list content. In the class, they modify their task list content under the guidance of the postgraduate teaching assistants, and repeat the process of their dialogue with AI. They discuss the problems that always exist in the process and the advantages and disadvantages of AI’s answers. Finally, the teacher gives guidance.
3. All researchers are instructors and assistants of the course, everybody is very familiar with course design and implementation, and has systematically taught or studied relevant content of qualitative research in nursing research. They have mastered basic research methods and interview skills, and the interview is conducted in a specially borrowed conference room with a quiet and undisturbed environment.

### Rigor and trustworthiness

1. Data were collected from undergraduate nursing students with different characteristics using a maximum difference sampling strategy to obtain more comprehensive findings.
2. After transcribing the interview recordings, we return them to the participants for verification to ensure the authenticity of the data to enhance the rigor and credibility of research.
3. When conducting data analysis, the crowdsourcing method was adopted, with two researchers working together to analyze and code interview transcripts, write memos and reflections, clarify the coding and topic content, discuss with other researchers when there are differences of opinion, and verify the participants to ensure the accuracy and credibility of the analysis results.

### Ethical considerations

A more direct awareness of the complexities of ethics while using AI in health professions education is required [15]. This study is a research and practice project on undergraduate education and teaching reform at the autonomous region level of Ningxia Medical University. The research conducted has been approved by the Ethics Committee of Ningxia Medical University. Researchers were careful about not extending the interview time and paying attention to the students’ reactions. To protect the privacy of participants, and all the collected data, including audio recordings and transcripts, were securely stored and accessible only to the researchers.

## Results

### Participant characteristics

All Fourteen students (nine females and five males) took part in this study, general information of participants can be found in Table 2.

**Table 2:** Basic characteristic of participants

Number	Class	Gender	Student cadres	Project issue
1	1	female	No	PI 2
2	1	female	Yes	PI 3
3	1	male	No	PI 1
4	3	male	No	PI 2
5	3	male	No	PI 2
6	3	female	Yes	PI 4
7	3	female	Yes	PI 6
8	3	female	No	PI 3
9	2	female	No	PI 5
10	2	male	Yes	PI 6
11	2	female	No	PI 2
12	2	female	No	PI 4
13	2	female	No	PI 1
14	1	male	No	PI 6

Project issue

PI 1: How is the research and innovation ability of undergraduate nursing students?

PI 2: Is the research and innovation ability of undergraduate nursing students related to their background in humanities and sciences?

PI 3: What is the impact of postgraduate entrance examination motivation on the research and innovation ability of undergraduate nursing students?

PI 4: Which scale is more suitable for evaluating the research and innovation ability of undergraduate nursing students?

PI 5: What is the experience of undergraduate nursing students participating in artificial intelligence + project task driven learning?

PI 6: How effective is the combination of artificial intelligence and project task driven teaching method in nursing research?

### Key themes

**Fig. 2:**
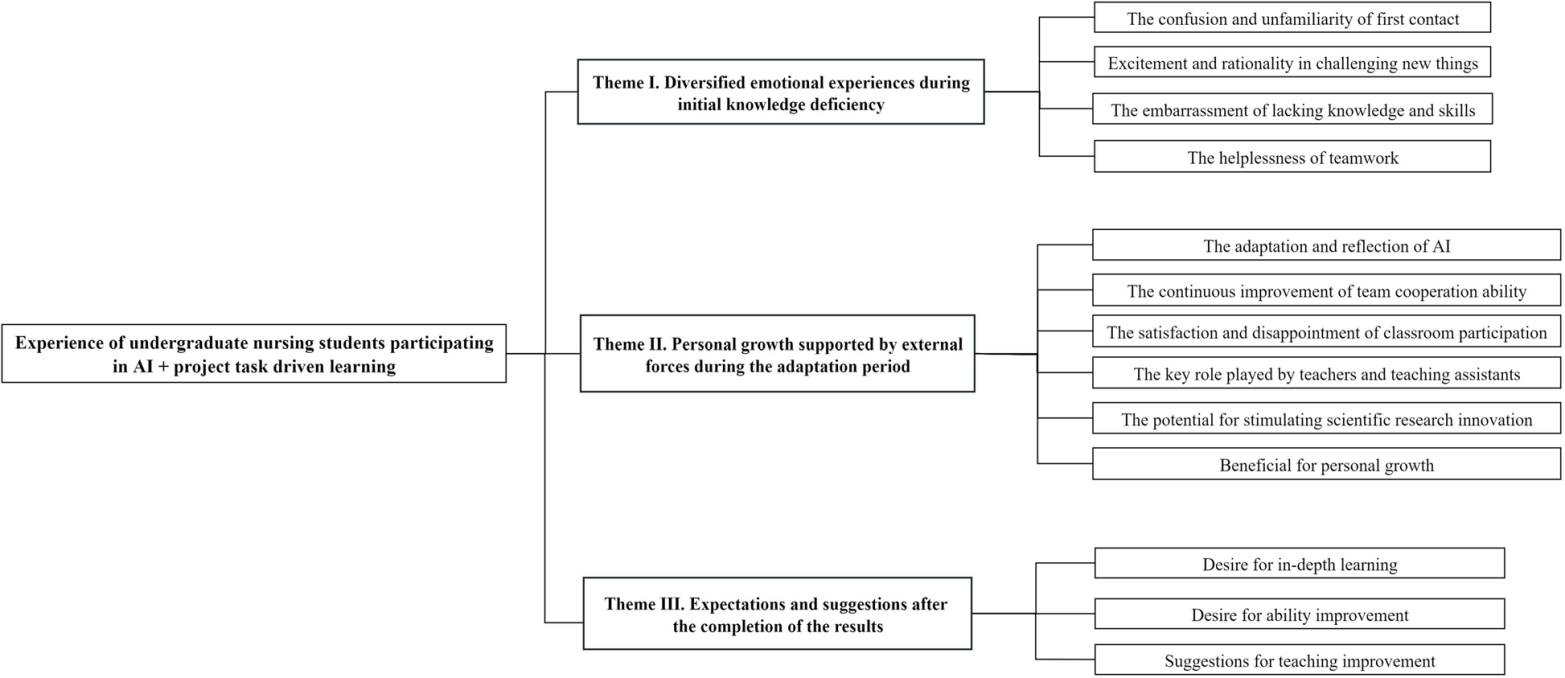
Themes and subthemes

According to interview data, the results are presented in the three themes and thirteen subthemes. Refer to Fig. 2 for details.

### Diversified emotional experiences during initial knowledge deficiency

#### The confusion and unfamiliarity of first contact

Encountering AI + project task driven learning for the first time, many students feel unfamiliar and confused. Faced with new fields and technologies, they feel unfamiliar and don’t know how to start.

*“It feels like a primitive person has entered modern society.”(Student B)*.

*“At the beginning, it may be unfamiliar and I don’t know how to start.”(Student F)*.

*“Suddenly coming into contact with this thing feels like I’m caught off guard.”(Student K)*.

#### Excitement and rationality in challenging new things

Faced with relatively new things, students often feel excited and stimulate their interest and motivation in learning.

*“Let’s be more interested in this course!”(Student A)*.

*“The teaching method is different from before… I am more interested in learning this course.”(Student I)*.

However, during the learning process, they did not rely entirely on AI, and maintained rational thinking.

*“It’s not about AI for research; it’s about utilizing it and not letting it dominates our thoughts.”(Student B)*.

*“It’s still about me being the main focus… It’s just an auxiliary type.”(Student H)*.

#### The embarrassment of lacking knowledge and skills

Students need to have a conversation with AI to complete project tasks. Although AI can intuitively help answer questions, after receiving answers, students need to apply professional knowledge to understand and also need to use computers to complete course task sheets. During this process, students express a lack of relevant knowledge and computer skills.

*“I don’t have a thorough understanding of the knowledge in that chapter of the textbook… I’m not very proficient in operating computers and so on.”(Student D)*.

*“It’s also because I’m not academically proficient and don’t quite understand what it has listed… I still lack professional knowledge.”(Student H)*.

*“My personal difficulty is… when searching for information; I am not very familiar with computer.”(Student A)*.

#### The helplessness of teamwork

This course is the first attempt to use AI combined with project tasks for teaching. When forming groups, we did not continue the learning groups already formed in other courses. Instead, we grouped students based on their classroom and daily performance, as well as their individual abilities. This also poses certain challenges to the ability of teamwork.

*“Moreover, the grouping is also chaotic, and the group members have not adapted well and are not well coordinated enough.”(Student D)*.

*“When there is no leader in the entire team… there is a certain lag… there is no way to continue.” “Actually, everyone has different ideas and is talking about themselves.”(Student L)*.

### Personal growth supported by external forces during the adaptation period

#### The adaptation and reflection of AI

As project tasks continue to advance, students gradually become familiar with the application of AI and begin to adapt to the process of using AI to complete corresponding tasks.

*“For example, those who need literature search in the past few days… using the current AI is very convenient.”(Student F)*.

*“Having it answer the same question multiple times can make it more accurate, and combining it with what is in the book can make it similar.”(Student M)*.

Some students expressed their dependence on AI, which also led them to start self-reflection while using it to solve problems.

*“Those who know or don’t will ask it… I’m starting to rely on AI.”(Student N)*.

*“I feel like after asking AI, its answers are also quite mechanical, a bit rigid.”(Student L)*.

#### The continuous improvement of team cooperation ability

With the continuous deepening of course learning, the form of group cooperative learning is constantly maturing. When completing project tasks through team cooperation, students are also learning, communicating, and collaborating with each other, which can better leverage their respective strengths.

*“Basically, it’s done together by everyone… it’s discussed with the group.”(Student C)*.

*“In group discussions, everyone can discuss… Working with the group can also enhance teamwork skills.”(Student D)*.

*“It feels like everyone is more united and proficient in doing this.”(Student E)*.

#### The satisfaction and disappointment of classroom participation

During the process of participating in the classroom, students sometimes feel satisfied and sometimes feel lost. Satisfaction stems from the recognition of completing group tasks and the joy of matching with the teacher’s answers.

*“Then when everything is completed in the end, I feel quite accomplished.”(Student D)*.

*“During class, when the teacher says the answer and it matches mine, I feel happier.”(Student J)*.

However, disappointment stems from insufficient knowledge reserves and a large gap between the group’s written content and the teacher’s explanation.

*“When I finish the task… I feel like I wrote it well… but when the teacher gave feedback in class, I felt like I was being hit.”(Student I)*.

#### The key role played by teachers and teaching assistants

During the project implementation process, teachers and teaching assistants played a crucial guiding role. The teaching staff provided students with rich learning resources, and timely answered their questions during the course implementation. The teaching assistants familiarized themselves with the research content in advance based on the project problems of their own group. In class, they guided students to think critically and actively participated in classroom discussions.

*“It feels that the teacher is very responsible… indicating that the teacher attaches great importance to us.”(Student B)*.

*“The annotations given by the senior sister clearly indicate the direction we should study… that is the train of thought… and then we will start the second round along the train of thought…”(Student C)*.

*“And there is also the assistance provided by the senior sister and graduate student senior sister.”(Student A)*.

#### The potential for stimulating scientific research innovation

With the continuous deepening of research projects, students have continuously put forward their new perspectives and insights while completing project tasks, demonstrating potential in scientific research innovation.

*“If we start researching and designing this whole piece now, it feels a bit systematic and holistic, and then it seems more abundant than our previous knowledge.”(Student D)*.

*“Through our investigation… it promotes our technological innovation ability.” (Student H)*.

*“I think the combination of AI and this project has mainly cultivated my interest in learning and a scientific research mindset for me.” (Student J)*.

#### Beneficial for personal growth

After experiencing a series of emotional experiences in the early stages, students experience personal growth during the adaptation period. These experiences have positive implications for their future learning and personal development.

*“You need to think from all aspects to broaden your thinking, and it seems like you have a career plan and a deeper understanding for the future.”(Student D)*.

*“You can use some basic software to expand your database and information.”(Student J)*.

*“It can better consolidate what you have learned in class… and improve your ability to search for literature.”(Student I)*.

### Expectations and suggestions after the completion of the results

#### Desire for in-depth learning

After a period of systematic learning, students have gradually adapted to the learning method of combining AI with project tasks. After the initial confusion and mid-term personal growth, undergraduate students gradually began to master the learning skills under this mode, and gradually developed a desire for further learning. Most students expressed a desire to learn from multiple aspects and be consciously prepared before class, proactively seek ways to solve one’s own difficulties.

*“I plan to study computer knowledge well during the vacation.” (Student A)*.

*“Ability to write a proposal has been improved, and I hope to further learn relevant research methods.” (Student F)*.

#### Desire for ability improvement

The thesis related to project tasks for students in the final stage has formed a prototype, and their ability to communicate with AI and research abilities have been improved. On the basis of completing course tasks, they have developed a desire to further enhance themselves.

*“I have gained a deeper understanding of this course… I have further improved my skills in writing papers and reading literature.”(Student D)*.

*“At first, I found it difficult to get started with, but I gradually understood it. I hope to further improve my ability to use AI.”(Student G)*.

*“I will improve the quality of my graduation thesis in my future senior year.” (Student J)*.

#### Suggestions for teaching improvement

During the process of participating in the classroom, students not only improve their research skills and learning abilities, but also raise some questions and suggestions for the teacher’s classroom content and teaching arrangements.

*“I suggest extending the class hours a bit… I hope we can delve deeper into this teaching process in the future.”*(*Student B)*.

*“Practice is the most crucial… I think this aspect should be slightly strengthened.”(Student C)*.

*“Then there will be a check in class or a small questioning session before class.”(Student I)*.

## Discussion

### Dynamic evaluation of changes in student learning experience after integrating AI into teaching, providing corresponding support

There is currently limited research on the application of generative AI in teaching practice due to that the implementation of it in education was implemented at the end of 2022 [16]. Anne et al. summarized the ideas of primary school students and teachers regarding the use of AI in their research [17]. It is reported by Gwo-Jen Hwang that most of the studies on AI in nursing focused on evaluating the performance of AI tools and systems, and issues related to the cognition, affect, skills, and behaviors of the research subjects have been less frequently discussed [18]. This study shown that the learning experience of nursing undergraduate students in the process of participating in AI + project task driven learning is not static, but presents a dynamic and changing process with the continuous deepening of course learning.

At the beginning of being exposed to AI and project tasks, most students feel unfamiliar and confused. In the process of completing project tasks, it is not only necessary to have a dialogue with AI to obtain relevant information, but also to mobilize professional knowledge to judge and understand the above information. Finally completing the tasks of their own group, students feel embarrassed due to a lack of professional knowledge and computer skills. In addition, the newly formed group cooperation has not yet been established, at this stage, they have the most complex emotional experience. Students are able to combine their thinking with AI to complete tasks during adaptation stage, which is different from blind reliance on generative AI tools [19]. This may be due to the help of teachers and assistants. Although there may still be conflicting emotional experiences in the class, most students express that their research ideas are gradually becoming clear and interest in scientific research innovation is also increasing. At the end of the period, they completed the course graduation thesis as scheduled utilizing AI and exerting self-initiative. While completing the project tasks, students also improved basic research abilities and developed a strong interest in the learning approach driven by AI + project tasks. At the same time, based on a desire for in-depth learning and a vision for the future, suggestions for further improvement have also been put forward for the involvement and arrangement of teachers in the class. Under the education philosophy of “student-centered”, teachers should focus on understanding of students’ knowledge level, learning habits, etc., keep track of their learning situation at any time, and accurately grasp the learning needs of students at different stages. In the initial stage, teachers could provide students with clear and explicit guidance on the integration of AI and teaching courses, helping them overcome various emotional difficulties in the early stages. The adaptation period mainly aims to guide students in a timely manner in the process of classroom participation, and assist them in effectively responding with available resources. The end period can encourage students to actively explore the use of AI in future courses, while also conducting a thorough analysis of the learning situation, timely adopting student suggestions, and further optimizing course design. In this series of dynamic changes, the most important thing is that teachers should demonstrate to students the operation methods and usage responsibilities of AI through practical applications, which can enable students to grasp the technology and help dispel the fear of the unknown [20].

### Strengthen positive guidance and guide students to use AI correctly

ChatGAi can be used to provide educational resources and support to students in areas where access to human teachers is limited. This study indicated that students can utilize AI for more efficient learning, but at the same time, it can also lead to excessive dependence on it. This is consistent with the concerns of other research and may lead to flattening and fragmentation of the learning process, unbeneficial to systematic learning and in-depth thinking [5]. Language generation tool represented by ChatGAi can serve as an assistant for students and educators, but it cannot replace human intelligence or replicate the complexity of human thinking [21]. For nursing students who will mainly engage in clinical or nursing research related work in the future, teachers are supposed to strengthen positive guidance for students to use AI, and enable them to fully understand the advantages and limitations of relevant tools before they use them independently. Attention should be paid to cultivating students’ creativity, collaboration, communication, and critical thinking, cultivating their AI literacy, exploring effective ways to cultivate their digital literacy and AI literacy, and fundamentally solving the potential risks and negative impacts in the application process of AI technology [11]. In addition, according to the relevant requirements of government departments for generative AI services, students should be guided to comply with laws and administrative regulations, respect social morality and ethical ethics, always adhere to socialist core values, and use AI correctly [22].

### Improve teaching design, enhancing the enthusiasm of nursing undergraduate students to participate in introducing AI into classroom teaching mode learning

Integrating AI and education will lead to a new education development model and promote better education [23], including medical education. In order for medical educators to be properly prepared for AI, teachers need to have at least a fundamental knowledge of AI in relation to learning and teaching [24]. Choosing appropriate teaching methods and organizing effective teaching activities through media, as well as conducting certain teaching reflections and evaluations, is crucial for teaching activities [25]. The course content in our study is the first attempt to implement it. Although nursing graduates have gained some benefits during the completion of project tasks, there are also some problems such as excessive dependence. At the same time, students have also put forward relevant suggestions for the course implementation. Teachers can further improve the teaching plan based on the completion of the course and the suggestions provided by students, and continuously improve the teaching quality in subsequent courses.

## Conclusions

This study explored the experiential changes of nursing undergraduate students at different stages of participating in AI + project task driven learning through qualitative study. The final study found that students participating in class teaching with AI experienced diverse emotional experiences from the initial stage to the adaptation stage, and then to the end stage. Initially, they exhibited feelings of confusion, excitement, embarrassment, and helplessness. During the adaptation period, students gradually overcome difficulties and achieve personal growth with external support. At the end of the period, students developed a desire for in-depth learning after the birth of their achievements, developed a longing for self-improvement, and provided suggestions for teaching. Nursing educators need to pay attention to the changes in the learning experience of students after integrating AI into teaching, effectively carry out class teaching activities, and strengthen positive guidance to guide students to use AI correctly. In the context of the future trend of integrating AI into education and teaching, the quality of AI participation in undergraduate teaching should be improved.

### Implications

Our study explored the use of AI + project task driven learning in Nursing Research course to incorporate the dialogue between nursing undergraduate students and artificial intelligence into nursing teaching, and describes the learning experiences of students. This is beneficial for the further application of AI in teaching and provides reference for the further application of AI in medical and nursing education.

### Limitations

This study only explored the changes in the learning experience of nursing undergraduate students after applying AI + project task driven learning to the Nursing Research. The integration of other courses with AI needs further exploration.

## Data Availability

The raw data required to reproduce the above findings cannot be shared at this time due to ethical reasons.
